# Circulating Zinc-α2-glycoprotein levels and Insulin Resistance in Polycystic Ovary Syndrome

**DOI:** 10.1038/srep25934

**Published:** 2016-05-16

**Authors:** Yerui Lai, Jinhua Chen, Ling Li, Jingxia Yin, Junying He, Mengliu Yang, Yanjun Jia, Dongfang Liu, Hua Liu, Yong Liao, Gangyi Yang

**Affiliations:** 1 Department of Endocrinology, the Second Affiliated Hospital, Chongqing Medical University, 400010 Chongqing, China; 2 The Key Laboratory of Laboratory Medical Diagnostics in the Ministry of Education and Department of Clinical Biochemistry, College of Laboratory Medicine, Chongqing Medical University, 400010 Chongqing, China; 3 Department of Pediatrics, University of Mississippi Medical Center, Jackson, MS, USA; 4 Department of Endocrinology, Armed Police Hospital of Chongqing, 400061 Chongqing, China

## Abstract

The aim of study was to assess the relationship between zinc-α2-glycoprotein (ZAG) and androgen excess with insulin resistance in polycystic ovary syndrome (PCOS) women. 99 PCOS women and 100 healthy controls were recruited. Euglycemic-hyperinsulinemic clamp (EHC) was preformed to assess their insulin sensitivity. Circulating ZAG was determined with an ELISA kit. In healthy subjects, circulating ZAG levels exhibited a characteristic diurnal rhythm in humans, with a major nocturnal rise occurring between midnight and early morning. Circulating ZAG and M-value were much lower in PCOS women than in the controls. In all population, overweight/obese subjects had significantly lower circulating ZAG levels than lean individuals. Multiple linear regression analysis revealed that only M-value and the area under the curve for glucose were independently related factors to circulating ZAG in PCOS women. Multivariate logistic regression analysis showed that circulating ZAG was significantly associated with PCOS even after controlling for anthropometric variables, blood pressure, lipid profile and hormone levels. The PCOS women with high ZAG had fewer MetS, IGT and polycystic ovaries as compared with the low ZAG PCOS women. Taken together, circulating ZAG levels are reduced in women with PCOS and ZAG may be a cytokine associated with insulin resistance in PCOS women.

Polycystic ovary syndrome (PCOS) is characterized by elevated circulating androgen levels, chronic anovulation, and polycystic ovaries^1^. In addition to oligomenorrhea and hyperandrogenism, these women have profound insulin resistance (IR) and alterations in β-cell function^2^,^3^,^4^. Obesity, particularly abdominal obesity, exacerbates the reproductive and metabolic dysfunction (MetS)^4^,^5^. Women with PCOS have also increased incidence of metabolic syndrome, impaired glucose tolerance (IGT), and type 2 diabetes mellitus (T2DM) compared with that in control subjects^6^,^7^. Therefore, a reproducible, accurate marker of IR that predicts outcomes and therapeutic responses would assist clinical management of PCOS. In the recent past, the role of adipose tissue as both an inflammatory mediator and endocrine organ has raised interest in the academic community^8^,^9^. Adipokines, adipose derived factors such as leptin, adiponectin and resistin, have been shown to modulate insulin sensitivity and appear to play an important role in the pathogenesis of IR^10^,^11^.

Zinc-alpha-2-glycoprotein (ZAG) is a 41-kDa glycoprotein assigned to the Major Histocompatibility Complex (MHC) class I family of proteins^12^, and is a soluble protein first identified in human blood, representing 0.2% of total serum protein^13^. The biological functions of ZAG are not completely known, but it has been shown that ZAG is a novel adipokine and that its expression in adipose tissue is down-regulated in obese subjects^14^. It has also been reported that ZAG contributes to the control of body weight and induces lipolysis in adipocytes^15^. ZAG-deficient mice are susceptible to weight gain when fed a high fat diet, which is associated with decreased lipolysis, unresponsive to β3- adrenoreceptor agonists^15^. Importantly, Balaz *et al*. reported that silencing ZAG resulted in reduced adiponectin (ADI), insulin receptor substrate-1(IRS-1) and glucose transporters-4 (GLUT4) gene expression in primary human adipocytes indicating that ZAG plays an important role in modulating whole-body and adipose tissue insulin sensitivity^16^. Very recently, we have shown that circulating ZAG levels are lower in patients with newly diagnosed T2DM than in healthy subjects and are positively correlated with ADI, and inversely with body mass index (BMI), waist-to-hip ratio (WHR), and homeostasis model assessment of insulin resistance (HOMA-IR), further suggesting that ZAG may be an adipokine associated with IR^17^. However, to date, no prior studies have demonstrated circulating ZAG levels or their relationship to IR in a large population with PCOS. Therefore, the aims of this study were to compare circulating ZAG levels of PCOS women and control subjects and to investigate the association of ZAG with IR, metabolic syndrome, and hyperandrogenemia.

## Results

### Anthropometric, hormonal and metabolic parameters in study subjects

Clinical, anthropometric, and endocrine characteristics in normal and PCOS women are listed in Table 1. The normal and PCOS subjects were similar in age, diastolic blood pressure (DBP), triglycerides (TG), free fatty acid (FFA), homeostasis model assessment of β cell secretion (HOMA-β), progestogen (PROG), follicle-stimulating hormone (FSH), estradiol (E2) and dehydroepiandrosterone sulfate (DHEA-S). BMI, WHR, the percentage of body fat (FAT%), systolic blood pressure (SBP), total cholesterol(TC), low-density lipoprotein cholesterol (LDL-C), fasting blood glucose (FBG), fasting insulin (FIns), HbA1c%, the area under the curve for glucose (AUC_glucose_), the area under the curve for insulin (AUC_insulin_), HOMA-IR, prolactin (PRL), luteinizing hormone (LH), total testosterone (TEST), and free androgen index (FAI) were higher, whereas M-value and sex hormone binding globulin (SHBG) were lower in the PCOS women than in the controls (*P* < 0.05 or *P* < 0.01; Table 1).

### Circadian rhythm of circulating ZAG levels in healthy subjects

**Figure 1:**
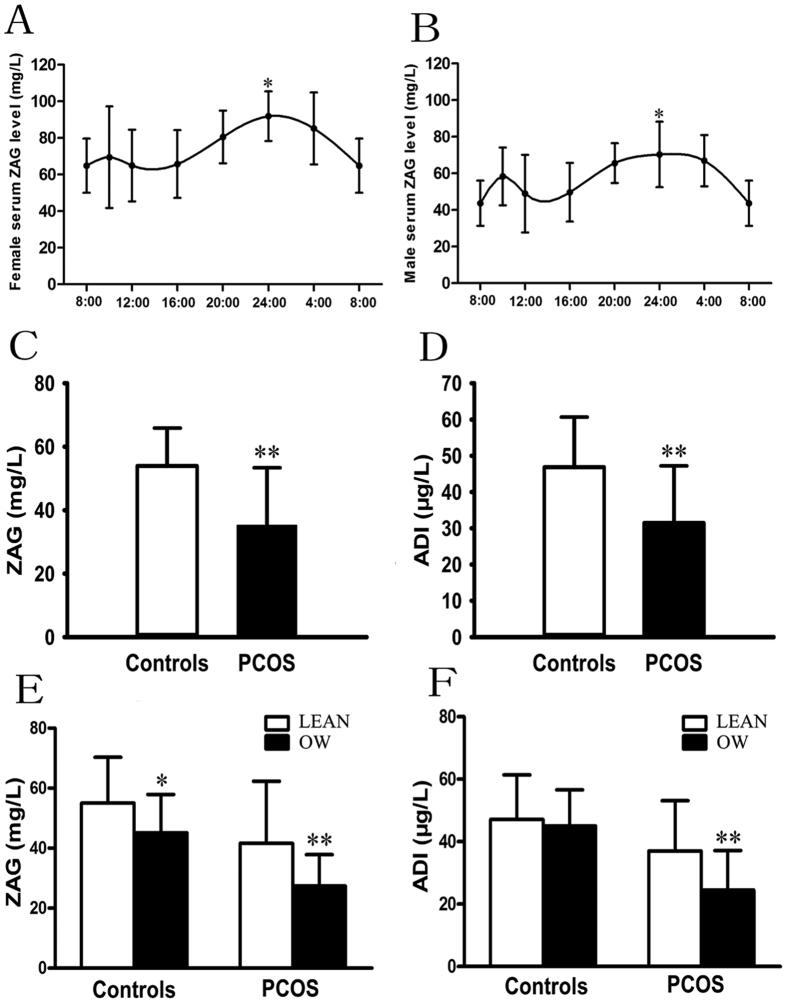
Circulating ZAG and ADI levels in study population. (**A**) Circadian circulating ZAG variations in female subjects. (**B**) Circulating ZAG levels in male subjects. (**C**) Circulating ZAG levels in PCOS and healthy women. (**D)** Circulating ADI levels in PCOS and healthy women. (**E**) Circulating ZAG levels according to lean or overweight in PCOS and healthy women. (**F**) Circulating ADI levels according to lean or overweight in PCOS and healthy women. LEAN : BMI < 25 kg/m^2^ and overweight/obese (OW): BMI ≥ 25 kg/m^2^; Values are given as means ± SD. vs. controls or normal-weight: ^*^P < 0.05, ^**^P < 0.01.

10 healthy subjects were provided with a standardized breakfast (0700), and their blood samples were collected from 0800 on day 1 to 0800 on day 2 to determine whether circulating ZAG is regulated by circadian-related factors. The result showed that a significant increase in circulating ZAG was observed at midnight in both male and female subjects. ZAG started to rise at 20:00 hr, peaked at 24:00 hr, and then decreased to nadir at 8:00 hr (*vs.* 8:00 hr, *P* < 0.05, Fig. 1A,B).

### Circulating ZAG level and its association with anthropometric, hormonal, biochemical parameters in study subjects

**Figure 2:**
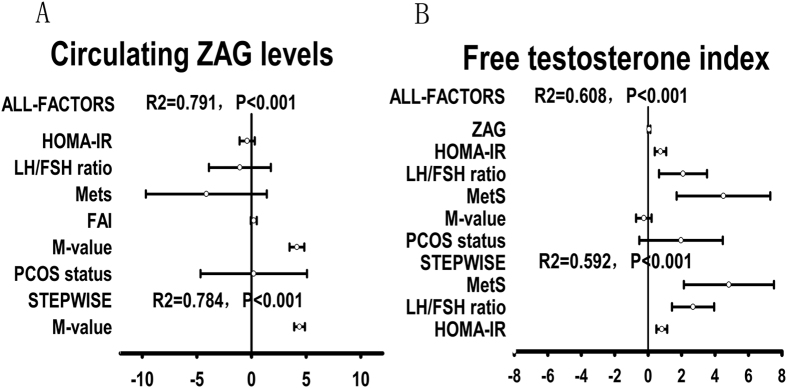
All factors and stepwise (probability for entry ≤0.05, probability for removal ≥0.10) multiple regression analyses of the circulating ZAG and FAI in all study population. The circles correspond to the regression coefficients (β) and the error bars indicate the 95% confidence interval of β. R2 = coefficient of determination. (**A**) The main predictor of circulating ZAG levels was M-value; (**B**) The main predictor of FAI levels was Mets, LH/FSH ratio and HOMA-IR.

Circulating ZAG levels are normally distributed in the two groups, ranging from 20.22 to 93.64 mg/L in control subjects and ranging from 11.27 to 83.5 mg/L in PCOS women. As shown in Fig. 1C, fasting ZAG levels were significantly lower in PCOS women than in normal women (35.25 ± 18.32 *vs.* 53.86 ± 15.31mg/L; *P* < 0.01). In addition, fasting ADI levels were also significantly lower in women with PCOS than in the controls (31.49 ± 15.93 *vs.* 46.94 ± 14.02 μg/L; *P* < 0.01; Fig. 1D). Circulating ZAG levels decreased 1.53-fold in PCOS women compared to the control subjects, whereas circulating ADI decreased 1.49-fold in PCOS women. In both normal and PCOS women, overweight/obese subjects (BMI ≥ 25 kg/m^2^) had significantly lower circulating ZAG levels than lean individuals (BMI < 25 kg/m^2^) (OW *vs.* lean: 45.16 ± 12.74 *vs.* 55.01 ± 15.32 mg/L for controls; 27.37 ± 10.47 *vs.* 41.66 ± 20.66 mg/L for PCOS; *P* < 0.05, Fig. 1E). In the healthy population, overweight women without menstrual irregularities also have significantly higher circulating ZAG levels than the PCOS women (45.17 ± 12.74 *vs.* 35.25 ± 18.32 mg/L; *P* < 0.01). In addition, overweight/obese PCOS women also had significantly lower circulating ADI levels than that of lean PCOS women (27.37 ± 10.47 *vs.* 41.66 ± 20.66 μg/L, *P* < 0.05; Fig. 1F). There is a trend of decrease in circulating ADI levels in healthy women with overweight/obese. However, this difference did not reach statistical significance (Fig. 1F). Pearson correlations showed that in PCOS women, ZAG negatively correlated with BMI, FAT%, TG, FIns, HbA1c, HOMA-IR, HOMA-β, AUC_glucose_, AUC_insulin_, and positively correlated with SHBG, M-value and ADI (Table 2). In normal subjects, ZAG negatively correlated with HbA1c, HOMA-β, AUC_glucose_, AUC_insulin_, and positively correlated with M-value and ADI (Table 2). All these correlations remained statistically significant after adjustment for age. Multiple linear regression analysis showed that only the M-value and AUC_glucose_ were independently related factors to circulating ZAG in PCOS women, whereas M-value, AUC_glucose_ and DHEA-S were independently related factors to circulating ZAG in normal subjects (Table 2). The multiple regression equations were: Y_ZAG-PCOS_ = 23.89 + 3.91X _M-value_ −0.74 AUC_glucose_ (R = 0.792, R^2^ = 0.627) and Y_ZAG-Normal_ = 18.5 + 4.02 X _M-value_ −0.71 X AUC_glucose_ + 0.02 X _DHEA-S_ (R = 0.797, R^2^ = 0.635). Multivariate logistic regression analysis showed that circulating ZAG levels were significantly associated with PCOS even after controlling for anthropometric variables, blood pressure, lipid profile and hormone levels (Table 3). When considering patients with PCOS and controls as a whole, regression analyses, including all-factor and stepwise models, showed that the main predictor of circulating ZAG concentrations was the M-value, whereas the main predictors of FAI levels were MetS, LH/FSH ratio, and HOMA-IR (Fig. 2). In addition, decreasing levels of ZAG showed a significant linear trend and were independently associated with PCOS, when concentrations were analyzed both by a row mean scores test and a Cochran-Armitage trend test (Table 4).

### Subgroup analysis

For subgroup analysis, high ZAG was defined by the unilateral 95% confidence intervals (ZAG ≥31.3 mg/L) in the 92 healthy women with normal weight. High M-vlue was defined by M-value ≥6.28^18^, and hyperandrogenemia was defined by FAI ≥5^19^. According to the high or low concentrations of ZAG, M-value, or FAI, PCOS women were further divided into subgroups. As shown in Table 5, BMI, HOMA-IR, AUC_glucose_ and AUC_insulin_ in PCOS women with a high ZAG level were significantly lower than in PCOS women with a low ZAG level, whereas the M-value, ADI and SHBG were significantly higher in the former (*P* < 0.05 or *P* < 0.01). Importantly, the PCOS subgroup with high ZAG had fewer MetS, IGT and polycystic ovaries as compared with the low ZAG subgroup (Table 5). PCOS women with high M-values had higher ZAG, ADI, SHBG levels and lower DHEA-S levels as well as fewer MetS, and IGT as compared with the low M-value subgroup. Comparison of hyperandrogenemic (FAI) and normoandrogenemic PCOS subjects showed that HOMA-IR, HOMA-β and DHEA-S were higher, whereas ZAG, SHBG and M-value were lower, and MetS and IGT were more in the former (Table 5).

### The predictive value of circulating ZAG in detecting PCOS, IR and MetS

**Figure 3:**
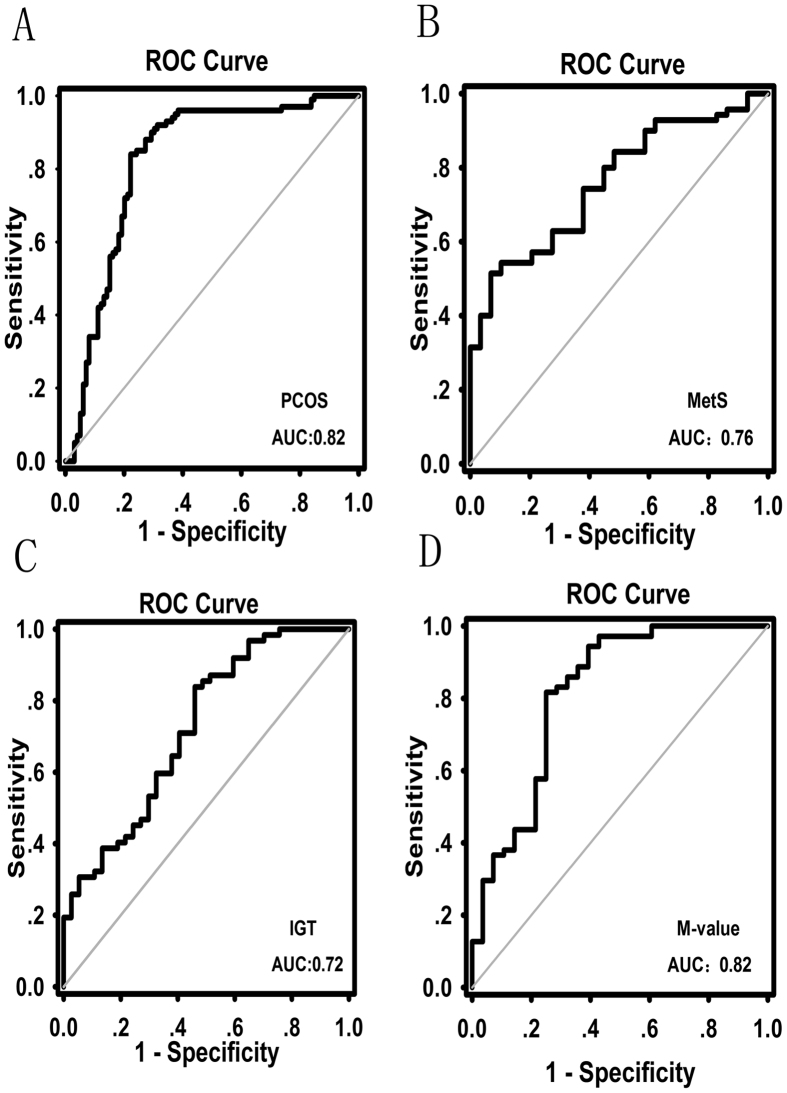
ROC curve analyses were performed for (**A**) the prediction of PCOS, (**B**) metabolic syndrome (MetS), (**C**) impaired glucose tolerance (IGT) and (**D**) M-value according to the ZAG levels.

To investigate the predictive value of ZAG for risk stratification of dysmetabolism in PCOS, MetS, IGT and IR (defined as M-value <6.28), we analyzed the receiver operator characteristic (ROC) curves of circulating ZAG. The ROC curve analyses revealed that the cutoff value for circulating ZAG to predict PCOS was 42.6 mg/L (sensitivity 84.0%, specificity 77.8%, and AUC 0.82; Fig. 3A). In PCOS patients, the cutoff value of the ZAG for predicting MetS was 35.7 mg/L (sensitivity 51.4%, specificity 93.1%, and AUC 0.76; Fig. 3B), for IGT 25.0 mg/L (sensitivity 83.9%, specificity 54.1%, and AUC 0.72; Fig. 3C), and for IR 38.3 mg/L (sensitivity 75.0%, specificity 81.7%, and AUC 0.82; Fig. 3D), respectively. In addition, we also analyzed the association of ZAG with hyperandrogenemia, anovulation and polycystic ovarian morphology by ROC curves. The results showed that ZAG was not a good predictor for other components involved in PCOS diagnosis (Supplemental Fig. 1A–C).

## Discussion

Recent comprehensive studies in rodents and humans provide convincing evidence of a link between IR and ZAG. However, human data have been inconsistent. In addition, it is also unknown about the circadian rhythm of circulating ZAG levels in healthy subjects. Here, our study provides the first evidence showing that circulating ZAG levels exhibit a characteristic diurnal rhythm in humans, with a major nocturnal rise occurring between midnight and early morning. This finding is in keeping with the hypothesis that ZAG may be a metabolic regulator in humans. A number of metabolic hormones, including leptin^20^, have been shown to exhibit a nocturnal rise, constituting an important mechanism for metabolic adaptation. For example, the nocturnal rise of leptin might be related to its appetite-suppressing effect during nighttime sleep^20^. Therefore, the nocturnal rise of ZAG may also be actively involved in appetite- suppressing effect during the night.

Notably, to date, no study has quantified circulating ZAG levels in patients with PCOS. It has also not been evaluated whether circulating levels of ZAG are changed in PCOS women in a similar way to other adipokines, such as ADI, and might, therefore, contribute to the pathogenesis of PCOS. PCOS is a known insulin-resistant state, and in the current human PCOS study, we have demonstrated on the basis of euglycemic-hyperinsulinemic clamp (EHC, M-value) that women with PCOS or overweight/obese are more IR than control subjects and have different circulating ZAG levels. In PCOS or overweight/obese women, circulating ZAG levels were significantly decreased. Furthermore, in PCOS subjects, ZAG levels correlated with other IR markers, such as HOMA-IR, M-value, and obesity related parameters, such as BMI, WHR and FAT%. These changes were similar to that of ADI, a known insulin sensitizer, in an insulin-resistant state, thus further suggesting ZAG is associated with IR and obesity.

The EHC is considered as the ‘gold standard’ measure of insulin sensitivity. EHC is also the method with the fewest drawbacks and is closest to the real measure of insulin sensitivity^21^. In the current study, we reported that circulating ZAG highly correlates with the insulin insensitivity assessed by EHC (M-value) in PCOS women. Unlike T2DM, PCOS women are young women who are IR, but have no severe glucose dysmetabolism. Hence, women with PCOS provide a useful model to investigate the relationship between ZAG and IR without the influence of severe glucose dysmetabolism. However, future studies are needed to explore whether ZAG is better than the usual biomarkers of IR in identifying subtle abnormalities.

In subgroup analysis, we further found that PCOS women with high ZAG had lower HOMA-IR and higher M-value. These women had also fewer MetS and IGT as compared with PCOS women with low ZAG. Therefore, these data further suggest that ZAG may be a useful marker of IR, MetS and glucose dysmetabolism.

Some data support a bidirectional relationship between hyperandrogenism and IR. *In vitro*, insulin may enhance LH-dependent ovarian androgen production, especially in theca cells from PCOS women^22^,^23^. *In vivo*, sustained hyperinsulinemia amplifies both GnRH- agonist induced ovarian steroidogenesis and ACTH-stimulated adrenal steroidogenesis^1^. Furthermore, insulin inhibits SHBG production in the liver, thereby increasing free androgen levels^24^. In addition, it was shown that testosterone administration may induce IR and changes in muscle fiber composition in ovariectomized rats^25^. Treatment with antiandrogens improved insulin sensitivity in hyperandrogenic women^26^. However, most studies relied on rough surrogate indices of insulin sensitivity, such as those based on glucose and insulin levels at fasting or after oral glucose tolerance test (such as glucose/insulin ratio, HOMA-IR). These indices correlate poorly with accurate gold-standard measures of insulin action-EHC. Since PCOS women with androgen excess are at higher risk of IR, liver disease, and subclinical atherosclerosis compared to PCOS patients with normal androgen levels^27^,^28^,^29^. It is important to identify those patients who have abnormal androgen constellations. FAI is preferable to testosterone as a marker of androgen excess in women with PCOS. Therefore, in the present study, PCOS women were further divided into high or low levels of FAI subgroups. We found that HOMA-IR was higher, ZAG and M-value were lower, whereas MetS and IGT were found more in PCOS women with high FAI levels compared with PCOS women with low FAI levels. These data give further support to the concept that androgens may play an important role in determining IR in PCOS women, although they do not allow us to establish the direction of their connection.

To determine the predictive value of ZAG for IR, PCOS, IGT and MetS, we analyzed the ROC curves of circulating ZAG and these metabolic phenotypes. The ROC curve analyses indicate that the ZAG might be a useful marker for the diagnosis of PCOS and for the prediction of MetS, IGT, and IR in PCOS women. We also determined the optimal cutoff value of the ZAG for identifying individuals with IR, IGT, MetS or PCOS in the Chinese population. However, the low sensitivity and specificity for MetS and IGT in ROC analysis probably indicate the heterogeneity of MetS and IGT in women with PCOS. In fact, PCOS is characterized by heterogeneity in phenotypic manifestations mainly related to reproductive and hormonal aberrations and the presence of metabolic disturbances, including IGT and MetS^30^,^31^. It has also been shown that the presence of MetS and elevated fasting insulin in adolescents had a poor correlation with IGT^32^.

In addition, we analyzed the association of circulating ZAG with the other components involved in PCOS diagnosis, such as hyperandrogenemia, anovulation and polycystic ovarian morphology by ROC curves. We found that ZAG was not a predictor for these components. Therefore, we believe that the association of ZAG with PCOS is due to the high prevalence of IR in these women. Therefore, the main strengths of this study are 1) it’s prospective design with inclusion of newly diagnosed PCOS women prevents pharmacotherapy and other confounding variables such as age and sex; 2) the association between ZAG and IR is investigated by EHC, a gold standard for evaluating insulin sensitivity; 3) importantly, the predictive values of ZAG for IR, PCOS, IGT and MetS are evaluated.

Our study has also some limitations. First, our cross-sectional design limits any firm conclusion about the possible causative role of ZAG in IR and PCOS. This would require longitudinal intervention studies and warrants future investigation. Secondly, our sample constituted entirely of Chinese women. Therefore extrapolation of these results to other ethnic groups should be undertaken with caution. In addition, the study was also limited by a relatively small sample, although the number of subjects included would provide more than 90% power to demonstrate associations at the conventional α < 0.05 level. Nonetheless, this study is sufficient to demonstrate novel associations of circulating ZAG with hormonal, metabolic parameters, and IR in PCOS women.

Overall, our findings demonstrate that decreased ZAG levels are a feature of IR and MetS, and hyperandrogenism may contribute to IR in PCOS women. We also found that the main predictor of circulating ZAG concentrations was M-value, whereas the main predictors of FAI levels were Mets, LH/FSH ratio, and HOMA-IR. However, further longitudinal and interventional studies are needed to clarify the clinical and pathophysiological significance of a decreased ZAG level in women with PCOS.

## Methods

### Study population

The PCOS group comprised 99 women who were referred to the Department of Endocrinology and Gynecology of the Second Affiliated Hospital of Chongqing Medical University from January 2013 to January 2014 due to menstrual irregularities. The enrolled women with PCOS were in good health and not suffering from chronic or acute diseases. The diagnosis of PCOS was based on the 2003 Rotterdam consensus (The Rotterdam ESHRE/ASRM-sponsored PCOS consensus workshop group) with at least two of the following features^1^: 1) oligo-amenorrhea or chronic anovulation; 2) clinical and/or biochemical hyperandrogenism; 3) ultrasound appearance of polycystic ovaries, after exclusion of other known causes of hyperandrogenemia and ovulatory dysfunction, including 21-hydroxylase deficiency, congenital adrenal hyperplasia, Cushing’s syndrome, androgen- secreting tumors, thyroid disease, and hyperprolactinemia. MetS was defined as three or more of the following: 1) BMI ≥25 kg/m^2^; 2) diabetes was confirmed or FBG ≥6.1 mmol/L and/or 2h- postprandial glucose (2h- BG) ≥7.8 mmol/L (IGT); 3) high blood pressure was confirmed or SBP/DBP ≥140/90 mmHg ; 4) triglyceride ≥1.7 mmol/L and/or high-density lipoprotein cholesterol (HDL-C) <0.9 mmol/L in male or HDL-C <1.0 mmol/L in female^33^. One hundred healthy women with regular periods and no hyperandrogenemia, hirsutism, or acne served as the control group, and they were studied during the follicular phase (progesterone <5 ng/ml). In this study, the proportion of MetS was 3% for the controls and 29% for PCOS, whereas the proportion of IGT was 2% for the controls and 37% for PCOS, respectively. Exclusion criteria for both groups included age >40 years, BMI >35 kg/m^2^, known cardiovascular disease, thyroid disease, neoplasms, smoking, diabetes, and renal impairment (serum creatinine 120 μmol/L). Oral contraceptives or other drugs involving in carbohydrate metabolism, if administered, were discontinued for at least 3 months before the study. All subjects gave their written informed consent before entering the study, which was conducted in accordance with the Declaration of Helsinki and approved by the ethical committee of the Second Affiliated Hospital of Chongqing Medical University.

### Oral glucose tolerance test (OGTT) and EHC

At 0800 h on the study days, after an 8–10 h overnight fast, an OGTT was performed on all subjects. These subjects ingested 75 g glucose, and venous blood was drawn at 0, 30, 60, and 120 min for the measurement of glucose and insulin.

EHC was performed on 99 women with PCOS and 100 healthy women as previously described^17^. Briefly, after an overnight fast, an iv catheter was placed in the antecubital vein to infuse insulin and glucose. Another catheter was placed retrograde in the dorsal vein of the contralateral hand for blood withdrawal. Regular human insulin (1 mU/kg/min) was infused for 2 h, and a variable infusion of 20% glucose was administered to maintain plasma glucose at the fasting level. During the procedure, plasma glucose levels were measured every 10 min to guide the glucose infusion. The rate of glucose disposal was defined as the glucose infusion rate (GIR) during the stable period of the clamp and was related to body weight (M value)^34^. Blood samples for ZAG and insulin measurements were obtained at fasting condition. All blood samples were centrifuged, and the separated serum or plasma was kept frozen at −80 °C until the time of the assay.

### The daily secretion study

In another separate sub-study, the healthy subjects (5 women and 5 men) aged 18–35 were admitted to the metabolic ward at 0600 and were provided with a standardized meal. Blood samples were drawn from an indwelling venous catheter in the forearm at 0800, 1000, 1200, 1600, 2000, 2400, and 0400 over a period of 24 h to investigate the daily ZAG levels. Blood was immediately centrifuged, and the plasma was separated and stored at −80 °C for measurement of ZAG.

### Biochemical and hormonal analysis

Blood samples were collected in the early-follicular phase (day 3 to 5 of the menstrual cycle) in the control group. Blood samples were collected after a spontaneous bleeding episode or upon first examination in PCOS women. Plasma glucose and HbA1c were measured by the glucose oxidase method and anion exchange high-performance liquid chromatography, respectively. Insulin was measured by radioimmunoassay using human insulin as standard (Institute of Atomic Energy, China). Free fatty acid (FFA) was measured with a commercial kit (Randox Laboratories, Antrim, U.K.). TC, HDL-C, LDL-C, and triglyceride were analyzed enzymatically using an autoanalyzer (Hitachi, Tokyo, Japan). Serum hormonal concentrations including LH, FSH, testosterone and Prog, PRL and E_2_ were measured with well-established electrochemi-luminescence immunoassay using COBAS E immunoassay analyzers (Roche Diagnostics GmbH). Total testosterone levels were measured by coated tube RIA (DiaSorin, S.p.A, Salluggia, Italy). DHEA-S and SHBG were performed using an automated analyzer (Abbott Architect; Abbott Laboratories, Abbott Park, IL). FAI was calculated as FAI = (testosterone/SHBG) ×100.

### Measurements of circulation ZAG and ADI

Circulating ZAG levels were determined with an ELISA kit obtained from Ray Biotech (Catalog #: EL-PRELIM, Beijing, China) following the manufacturer’s protocol with slight modification. Briefly, 100 μl human plasma was collected from subjects who had fasted overnight using EDTA-containing tubes, and samples were applied to the test wells along with ZAG standards of concentration 7.8–500 μg/ml. Then, 100 μl of specific biotin- conjugated anti-human ZAG was added to each well and incubated at 37 °C for 1 h. Each well was then washed three times with PBS containing 0.05% Tween-20 (pH 7.2–7.4). Colorimetric reaction was performed for 20 min with the use of horseradish peroxidase– conjugated streptavidin (Zymed, South San Francisco, CA) as substrate. Optical densities were measured at 450 nm. A calibration curve was constructed by plotting the absorbance values at 450 nm *vs.* the ZAG concentrations of the calibrators, and concentrations of plasma samples were determined by using this calibration curve. The limit of detection was 1.95 ng/mL, and intra-assay and inter-assay variations were <10% and <12%, respectively. The assay has high sensitivity and excellent specificity for detection of human ZAG with no significant cross-reactivity or interference. Human ZAG ELISA was performed in duplicate. If duplicates had 10% CV, the sample was repeated. Circulating ADI level was also measured by ELISA as we previously described^17^. The limit of detection was 1.102 ng/mL, and intra-assay and inter-assay variations were <8% and <10%, respectively.

### Anthropometric measurements

BMI was calculated as weight divided by height squared. The percentage of body fat (FAT %) was measured by bioelectrical impedance (BIA-101; RJL Systems, Shenzhen, China). The HOMA-IR = FIns (mU/L) × FBG (mmol)/22.5. HOMA-β = 20 × FIns (mU/L)/FBG (mmol) −3.5^35^. AUC_glucose_ and AUC_Insulin_ during the OGTT were calculated geometrically using the trapezoidal rule.

### Statistical analysis

The stratified Mantel Haenszel row mean score test was used to assess whether there was a change in the prevalence rate of PCOS and ordered categories of ZAG level was row mean scores differ on average across all subjects. Trend tests of PCOS incidences across the three categories of ZAG levels were conducted by Cochran-Armitage tend tests. Row mean score test and Cochran-Armitage trend tests were conducted using SAS software version 9.30 (SAS Institute Inc, Cary, NC), while the other statistical analyses were conducted using SPSS software version 19.0 (SPSS, Chicago, IL). Results are expressed as mean ± SD or median (interquartile range) unless stated otherwise. Variables with a non-normal distribution were transformed by logarithm or square-root before analysis. Independent sample *t* test was used in comparisons between two groups. Pearson correlation analysis was used to evaluate the relationship of ZAG with IR and other covariates. Multiple linear regression analysis using a stepwise method (probability for entry ≤0.05, probability for removal ≥0.10) for the introduction of independent variables was used to identify the main determinants of ZAG levels and FAI among the variables showing a statistically significant correlation in univariate analysis. Receiver operating characteristics (ROC) curves of ZAG levels were constructed to determine the optimal cutoff point for the prediction of PCOS, MetS, FAI and IR (assessed by HOMA-IR or M-values). *P* values < 0.05 (two-tailed) were considered statistically significant.

## Additional Information

**How to cite this article**: Lai, Y. *et al*. Circulating Zinc-α2-Glycoprotein levels and Insulin Resistance in Polycystic Ovary Syndrome. *Sci. Rep.* **6**, 25934; doi: 10.1038/srep25934 (2016).

## Supplementary Material

Supplementary Information

## Figures and Tables

**Table 1:** Main clinical features and circulating ZAG levels in PCOS and controls.

Group	PCOS	Controls	*P*-value
N	99	100	NS
Age (yr)	26.0 ± 4.6	25.6 ± 2.2	NS
BMI (kg/m2)	24.6 ± 4.8	20.5 ± 2.7	<0.01
FAT (%)	35.1 ± 9.8	26.7 ± 5.6	<0.01
WHR	0.9 ± 0.1	0.8 ± 0.1	<0.01
SBP (mmHg)	116.1 ± 10.5	109.1 ± 8.0	<0.01
DBP (mmHg)	76.9 ± 7.1	74.8 ± 7.8	NS
TG (mmol/L)^a^	1.30 (0.86–1.89)	0.80 (0.58–1.29)	NS
TC (mmol/L)	4.44 ± 1.03	3.85 ± 1.00	<0.01
HDL-C (mmol/L)	1.34 ± 0.65	1.17 ± 0.30	NS
LDL-C (mmol/L)	2.52 ± 0.82	2.17 ± 0.88	<0.01
FFA (umol/L)	0.60 ± 0.21	0.56 ± 0.29	NS
FBG (mmol/L)	5.02 ± 1.24	4.4 ± 0.45	<0.01
FIns (pmol/L)^a^	98.9 (61.3–149.1)	49.5 (42.5–62.0)	<0.01
HbA1c (%)	5.40 ± 0.55	5.17 ± 0.25	<0.01
AUC_glucose_	16.0 ± 4.6	12.0 ± 2.3	<0.01
AUC_insulin_^a^	198.7 (144.5–270.2)	109.0 (62.5–146.0)	<0.01
HOMA-IR^a^	3.0 (1.7–4.8)	1.4 (1.2–1.8)	<0.01
HOMA-β^a^	211.4 (131.1–326.4)	158.4 (116.8–278.5)	NS
M-value (mg/kg/min)	5.87 ± 2.87	10.11 ± 2.64	<0.01
ADI (μg/L)	31.49 ± 15.93	46.94 ± 14.02	<0.01
ZAG (mg/L)	35.25 ± 18.32	53.86 ± 15.31	<0.01
PRL (mIU/L)	371.3 ± 178.9	325.0 ± 98.1	0.02
PROG (nmol/L)	2.6 ± 0.8	2.7 ± 1.1	NS
LH (IU/L)	10.0 ± 6.4	4.9 ± 2.7	<0.01
FSH (IU/L)	7.5 ± 2.3	8.1 ± 1.9	NS
TEST (nmol/L)	2.9 ± 1.6	1.8 ± 0.8	<0.01
E2 (pmol/L)	231.8 ± 167.2	198.9 ± 102.0	NS
DHEAS (μg/dL)^b^	193.1 (156.5–249.5)	182.1 (140.6–216.8)	NS
SHBG (nmol/L)^a^	34.5 (22.4–50.6)	57.4 (41.9–75.3)	<0.01
FAI^a^	8.4 (4.8–12.5)	2.6 (1.8–5.1)	<0.01

PCOS, polycystic ovary syndrome; BMI, Body mass index; WC, waist circumference; HC, hip circumference; SBP, Systolic blood pressure; DBP, Diastolic blood pressure; WHR, Waist hip ratio; FAT%, Body fat %; FBG, Fasting blood glucose; FIns, Fasting plasma insulin; HOMA-IR, HOMA-insulin resistance index ; HOMA-β, HOMA- β cell Secretion index; TG, Triglyeride; TC, Total cholesterol; HDL-C, High-density lipoprotein cholesterol; LDL-C, Low-density lipoprotein cholesterol ; FFA, free fatty acid; HbA1c, Glycosylated hemoglobin; M-value, whole body glucose uptake rate; ZAG, Zinc-α2-glycoprotein; ADI, adiponectin; AUC_insulin_, the area under the curve for insulin; AUC_glucose_, the area under the curve for glucose; PRL, prolactin; PROG, progestogen; LH, luteinizing Hormone; FSH, follicle- Stimulating Hormone; TEST, total testosterone; E2, estradiol; DHEAS, dehydroepiandrosterone sulfate; SHBG, sex hormone-binding globulin. Free androgen index (FAI) = TEST (nmol/L)/SHBG (nmol/L) × 100. Values were given as means ± SD or median (interquartile Range)^a^,

Log transformed before analysis^b^,

Square-root transformed before analysis.

**Table 2:** Linear correlation and multiple regression analysis of variables associated with serum ZAG levels in study subjects.

Variable	Simple				Multiple			
	PCOS		Controls		PCOS		Controls	
	Estimate	P-value	Estimate	P-value	Estimate	P-value	Estimate	P-value
BMI	−0.44	<0.001	−0.20	NS	–	–	–	–
FAT (%)	−0.34	<0.001	−0.20	NS	–	–	–	–
WHR	−0.02	0.05	0.00	NS	–	–	–	–
TG^a^	−0.21	0.03	0.14	NS	–	–	–	–
SHBG^a^	0.21	0.03	0.11	NS	–	–	–	–
DHEAS^b^	0.04	NS	−0.02	NS	–	–	0.09	0.04
FIns^a^	−0.51	<0.001	−0.18	NS	–	–	–	–
HbA1C	−0.30	<0.001	−0.21	0.04	–	–	–	–
HOMA-IR^a^	−0.51	<0.001	−0.13	NS	–	–	–	–
HOMA-β^a^	−0.31	<0.001	−0.21	0.04	–	–	–	–
M-value	0.82	<0.001	0.56	<0.001	0.70	<0.001	0.72	<0.001
AUC_glucose_	−0.42	<0.001	−0.26	0.01	−0.16	<0.001	−0.15	<0.001
AUC_insulin_^a^	−0.45	<0.001	−0.23	0.02	–	–	–	–
ADI	0.40	<0.001	0.22	0.03	–	–	–	–

^a^Log transformed before analysis.

^b^Square-root transformed before analysis. In multiple linear stepwise regression analysis, values included for analysis were age, BMI, FAT%, SBP, DBP, WHR, M-value, ADI, AUC_glucose_, AUC _Insulin_, HOMA-IR, HbA1C, TG, TC, HDL-C, LDL-C, DHEAS, E2, LH, FSH, PRL, PROG, FAI.

**Table 3:** Association of circulating ZAG with PCOS in fully adjusted models.

Model adjust	PCOS		
	OR	95% CI	*P*
Age, SBP, DBP	0.939	0.919–0.960	<0.001
Age, SBP, DBP, BMI, WHR	0.952	0.929–0.976	<0.001
Age, SBP, DBP, BMI, WHR, lipid profile	0.953	0.929–0.978	<0.001
Age, SBP, DBP, BMI, WHR, lipid profile, Hormone parameters	0.962	0.932–0.993	0.015

Lipid profile include TG, TC, HDL, LDL, FFA; Hormone parameters include SHBG, DHEAS, E2, TEST, FSH, LH, PROG, PRL.

**Table 4:** Row Mean Scores and Cochran–Armitage Trend Test of the impact of circulating ZAG on PCOS prevalence.

	PCOS	
	χ^2^	*P*-value
Row Mean Scores Test	75.8873	<0.001
Cochran-Armitage Trend Test	−7.6948	<0.001

**Table 5:** Main characteristics of PCOS women, as subdivided according to the high or low levels of ZAG, M-value, or FAI.

	ZAG		M-value		FAI	
	Low (n = 49)	High (n = 50)	Low (n = 71)	High (n = 28)	Low (n = 28)	Hight (n = 71)
Age (y)	25 (23–30)	27 (24–30)	26 (23–30)	26 (24–29)	28 (25–30)	26 (23–29)
BMI (kg/m^2^)	25.9 (22.5–28.6)	23.2 (19.7–26.4)*	25.9 (23.3–28.7)	19.8 (17.9–22.8)^∇∇^	22.8 (18.7–26.6)	25.0 (22.1–28.4)^▾^
MetS	21 (42.86%)	8 (16.00%)**	28 (39.44%)	1 (3.57%)^∇∇^	3 (10.71%)	26 (36.62%)^▾^
IGT	24 (48.98%)	13 (26.00%)*	36 (50.70%)	1 (3.57%)^∇∇^	6 (21.43%)	31 (43.66%)^▾^
HOMA-IR	4.55 (2.52–5.67)	2.12 (1.56–3.07)**	4.10 (2.65–5.63)	1.55 (1.13–2.30)^∇∇^	2.22 (1.35–3.68)	3.54 (1.85–4.85)^▾^
HOMA- β	219.6 (155.6–356.5)	180.6 (108.5–291.0)	256.1 (167.1–376.9)	122.9 (90.8–182.3)^∇∇^	151.1 (103.1–304.5)	219.3 (149.7–359.5)^▾^
M-value	3.98 (3.35–5.35)	6.10 (5.28–9.07)**	4.32 (3.54–5.50)	8.90 (7.44–11.81)^∇∇^	5.78 (4.07–11.06)	5.10 (3.62–6.26)^▾▾^
AUC_glucose_ (mmol *h/L)	16.5 (14.5–19.2)	13.4 (12.5–15.6)**	16.1 (14.1–19.0)	13.0 (11.9–14.5)^∇∇^	14.5 (12.7–16.3)	15.7 (13.3–18.6)
AUC_insulin_ (mU*h/L)	243.5 (160.8–315.8)	169.7 (135.7–216.3)**	238.0 (179.3–298.2)	135.3 (106.9–156.2)^∇∇^	169.7 (129.5–217.8)	215.8 (152.4–294.9)^▾^
ZAG (mg/L)	23.8 (19.0–26.6)	42.1 (35.9–52.6)**	27.6 (21.9–35.3)	47.9 (32.2–65.8)^∇∇^	35.4 (24.9–61.9)	30.0 (23.7–39.8)^▾▾^
ADI (μg/L)	28.1 (13.6–36.5)	36.5 (25.7–40.3)**	29.1 (15.3–36.5)	38.4 (27.9–54.9)^∇∇^	35.3 (23.6–51.1)	31.1 (19.3–36.5)^▾^
LH/FSH ratio	1.06 (0.72–1.70)	1.39 (0.81–1.96)	1.15 (0.68–1.71)	1.29 (0.85–2.15)	0.94 (0.61–1.57)	1.27 (0.78–1.84)
SHBG	29.7 (19.7–42.2)	38.4 (27.9–59.0)*	31.0 (19.8–49.5)	49.5 (29.1–71.0)^∇∇^	59.8 (36.8–88.0)	29.6 (19.8–40.6)^▾▾^
TEST (noml/L)	2.76 (1.95–3.38)	3.02 (2.33–3.68)	2.88 (2.32–3.68)	2.74 (2.14–3.51)	2.08 (0.79–2.74)	3.13 (2.57–3.71)^▾▾^
FAI	9.8 (5.5–14.9)	6.8 (4.5–10.3)	9.2 (5.1–15.8)	5.4 (3.9–9.3)^∇∇^	3.3 (1.9–4.3)	10.2 (7.6–15.8)^▾▾^
DHEAS (μg/dL)	193.6 (155.1–254.0)	191.7 (156.2–249.9)	202.9 (157.6–261.3)	173.1 (150.5–217.9)^∇^	160.9 (116.6–210.3)	201.4 (171.1–261.3)^▾▾^
Polycystic ovaries	34 (69.39%)	25 (50.00%)*	44 (61.97%)	15 (53.57%)	17 (60.71%)	42 (59%)

High ZAG was defined according to the unilateral 95% confidence intervals (ZAG ≥31.3 mg/L) by the 92 healthy women. High M-vlue was defined by M-value ≥6.28. Hyperandrogenemia was defined by FAI ≥5. BMI, Body mass index; MetS, metabolic syndrome; IGT, impaired glucose tolerance; HOMA-IR, HOMA-insulin resistance index ; HOMA-β, HOMA- β cell Secretion index; AUC_glucoae_, the area under the curve for glucose; AUC_insulin_, the area under the curve for insulin; ZAG, circulating Zinc-α2-glycoprotein; ADI, adiponectin; LH, Luteinizing Hormone; FSH, Follicle- Stimulating Hormone ; SHBG, sex hormone-binding globulin; TEST, total testosterone; FAI, Free androgen index; DHEAS, dehydroepiandrosterone sulfate. Data are median (interquartile Range) or frequency (percent). nonparametric test was used in comparisons between two groups. *P < 0.05, **P < 0.01 compared with low ZAG group; ^∇^*P* < 0.05, ^∇∇^*P* < 0.01 compared with low M-value group; ^▾^*P* < 0.05, ^▾▾^*P* < 0.01compared with low FAI group.
