# Supplementary material for: Circulating Zinc-α2-glycoprotein levels and Insulin Resistance in Polycystic Ovary Syndrome
Source: Sci Rep. 2016 May 16;6:25934. doi: 10.1038/srep25934 (PMC4867572; doi:10.1038/srep25934)
Supplement: Supplementary Information [file srep25934-s1.pdf]

Circulating Zinc-α2-glycoprotein levels and Insulin Resistance in Polycystic Ovary Syndrome

Yerui Lai[1,+], Jinhua Chen[1,+], Ling Li[2], Jingxia Yin[1], Junying He[1], Mengliu Yang[1], Yanjun Jia[2], Dongfang Liu[1], Hua Liu[3], Yong Liao[4], Gangyi Yang[1,*]

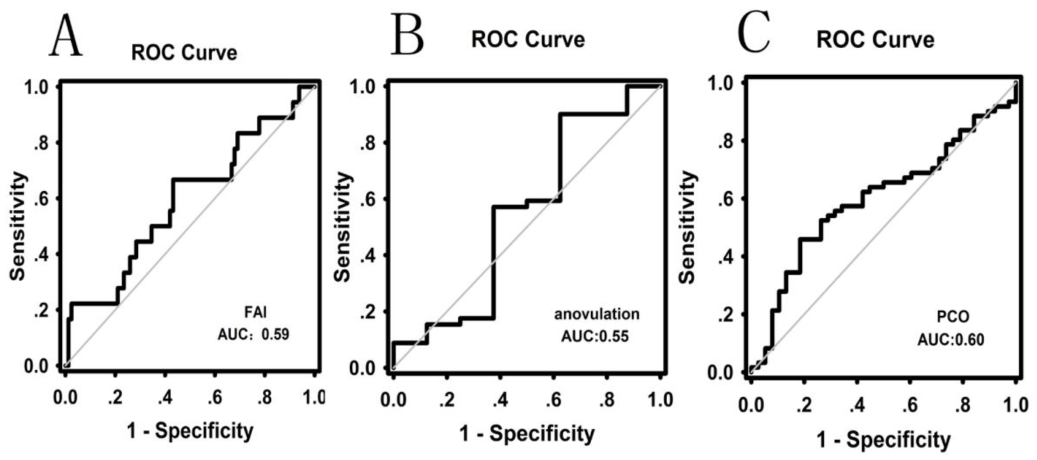

Supplemental Figure 1.ROC curve analyses were performed for **(A)** the prediction offree androgen index (FAI), **(B)** anovulation, and **(C)**polycystic ovaries (PCO)
